# Kisspeptin-10 Rescues Cholinergic Differentiated SHSY-5Y Cells from α-Synuclein-Induced Toxicity In Vitro

**DOI:** 10.3390/ijms23095193

**Published:** 2022-05-06

**Authors:** Christopher Simon, Tomoko Soga, Nafees Ahemad, Saatheeyavaane Bhuvanendran, Ishwar Parhar

**Affiliations:** 1 Brain Research Institute, Jeffrey Cheah School of Medicine and Health Sciences, Monash University Malaysia, Bandar Sunway, Selangor 47500, Malaysia; christopher.simon@monash.edu (C.S.); tomoko.soga@monash.edu (T.S.); bsaatheeyavaane.bhuvanendranpillai@monash.edu (S.B.); 2 School of Pharmacy, Monash University Malaysia, Bandar Sunway, Selangor 47500, Malaysia; nafees.ahemad@monash.edu

**Keywords:** dementia with Lewy bodies, amyloid-β, E46K mutant, C-terminal domain, GPR54, choline acetyltransferase, neuropeptide, neurodegeneration, neuroprotection, neurotoxicity

## Abstract

The neuropathological substrate of dementia with Lewy bodies (DLB) is defined by the inextricable cross-seeding accretion of amyloid-β (Aβ) and α-synuclein (α-syn)-laden deposits in cholinergic neurons. The recent revelation that neuropeptide kisspeptin-10 (KP-10) is able to mitigate Aβ toxicity via an extracellular binding mechanism may provide a new horizon for innovative drug design endeavors. Considering the sequence similarities between α-syn’s non-amyloid-β component (NAC) and Aβ’s C-terminus, we hypothesized that KP-10 would enhance cholinergic neuronal resistance against α-syn’s deleterious consequences through preferential binding. Here, human cholinergic SH-SY5Y cells were transiently transformed to upsurge the mRNA expression of α-syn while α-syn-mediated cholinergic toxicity was quantified utilizing a standardized viability-based assay. Remarkably, the E46K mutant α-syn displayed elevated α-syn mRNA levels, which subsequently induced more cellular toxicity compared with the wild-type α-syn in choline acetyltransferase (ChAT)-positive cholinergic neurons. Treatment with a high concentration of KP-10 (10 µM) further decreased cholinergic cell viability, while low concentrations of KP-10 (0.01–1 µM) substantially suppressed wild-type and E46K mutant α-syn-mediated toxicity. Correlating with the in vitro observations are approximations from in silico algorithms, which inferred that KP-10 binds favorably to the C-terminal residues of wild-type and E46K mutant α-syn with CDOCKER energy scores of −118.049 kcal/mol and −114.869 kcal/mol, respectively. Over the course of 50 ns simulation time, explicit-solvent molecular dynamics conjointly revealed that the docked complexes were relatively stable despite small-scale fluctuations upon assembly. Taken together, our findings insinuate that KP-10 may serve as a novel therapeutic scaffold with far-reaching implications for the conceptualization of α-syn-based treatments.

## 1. Introduction

Dementia with Lewy bodies (DLB) is an idiopathic neurodegenerative entity, pathologically lesioned by conspicuous degeneration of α-synuclein (α-syn)-rich cholinergic neurons in the senile brain [1,2,3]. Across pathological subtypes, the α-syn gene was incriminated in cholinergic neurons when the point mutation, E46K, was ascertained in kindreds with mixed phenotypes of parkinsonism and dementia that resembled DLB [4]. Beyond this pathogenetic continuum, co-immunoprecipitation experiments from human brain extracts further theorized a pervasive scenario for DLB pathogenesis encompassing heterologous collusion between α-syn and the amyloid-β (Aβ) peptide [5,6]. A 14-kDa monomer ubiquitously expressed in presynaptic cholinergic termini; the α-syn is inherently defined by an N-terminal domain (residues 1–60), an aggregation-prone non-amyloid-β component (NAC, residues 61–95), and a C-terminal region (residues 96–140) [7]. Given the sequence commonalities between α-syn’s NAC fragment and Aβ’s C-terminus [8], the prevailing view is that Aβ synergizes with the NAC portion and governs its transition into aggregates that exacerbates the dissemination of cholinergic pathology [9,10]. In a cross-seeding phenomenon that has been likened to that perceived in prionopathies [11], intracellular α-syn encased in exosomes are secreted to the extracellular milieu [12,13,14], thus enabling aggregative interactions with Aβ [15,16,17]. Since pharmacologically interceding this self-perpetuating pathocascade has yet to result in causal disease-modifying strategies, there is an unmet necessity to exploit novel targets for drug discovery. In this regard, the recent depiction of a hypothalamic-based neuropeptide, designated kisspeptin, in limbic and paralimbic brain regions underpinned an unprecedented dimension to the advent of neurotherapeutics in biological stress [18,19]. While the pathophysiological relevance of this occurrence was affiliated with neuropsychiatric behaviors well beyond the strict confines of the gonadotrophic axis [20,21], the corollary that it may be implicated in neurodegenerative pathologies is also widely believed. Ultimately, the unconventional fluctuations of kisspeptin-10 (KP-10) expression patterns and receptor densities in degenerative microenvironments were the features that led to its consideration as a surrogate neuroprotector [22,23]. Despite variable degrees of redundancy in substrate specificity, KP-10, which is biologically mediated by its cognate G protein-coupled receptor, GPR54 [24], inhibited the toxicity of extracellular Aβ via an action that GPR54 antagonists could not impede [22]. Paralleling these observations, ablation of the *KiSS-1* gene, which encodes the human kisspeptin, significantly enhanced the toxicity of Aβ, while *KiSS-1* overexpression in SH-SY5Y neurons established a cellular milieu that was resistant to Aβ toxicity [25]. Following this revelation, we hypothesized that exogenous administration of KP-10 could be neuroprotective against the characteristic deficiencies in DLB that result from the gradual degeneration of α-syn-laden cholinergic neurons. The progressive demise of cholinergic neurons has been previously ascribed to disease-linked determinants that augment α-syn’s mRNA expression [26,27] and aggregation propensity [28]. The transient transfection of α-syn in differentiated SHSY-5Y neurons has been modeled in a myriad of neuroprotective interventions to elucidate the biological implications of α-syn mRNA expression in disease causative mechanisms and anti-aggregation strategies [29]. In particular, prior reports have shown that differentiation of SH-SY5Y cells upon exposition to supraphysiological retinoic acid (RA) concentrations is integral for reflecting cytological homogeneity with choline acetyltransferase (ChAT)-expressing cholinergic neurons [30,31]. Thus, utilizing the SH-SY5Y human cholinergic neuroblastoma cellular model, the present study, for the first time, aimed to explore the extent to which KP-10 administration could confer neuroprotection against α-syn-induced toxicity. With this rationale in mind, cholinergic differentiated SH-SY5Y cells were genetically engineered to overexpress human wild-type or E46K mutant α-syn. To further refine the solvation dynamics of these interactions, a computational pipeline, amalgamating in silico docking, and molecular dynamics (MD) simulations were employed.

## 2. Results

### 2.1. RA-Differentiated SH-SY5Y Cells Establish a Cholinergic-like Phenotype

**Figure 1:**
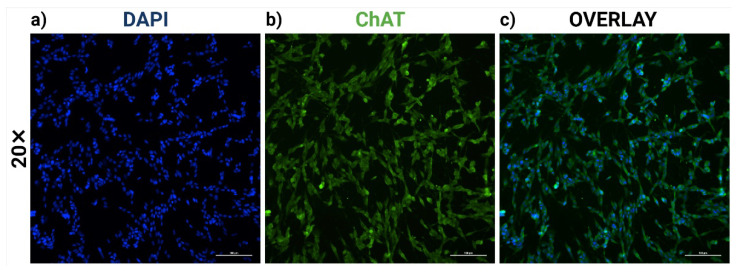
The immunocytochemical expression of choline acetyltransferase (ChAT) in retinoic acid (RA)-differentiated SHSY-5Y cells. Microscopic images were visualized at 20× magnification for (**a**) 4′, 6-diamidino-2-phenylindole (DAPI, blue; left), (**b**) ChAT (green; middle), and (**c**) Overlay (right). Scale bars, 100 μm. The percentage of ChAT-positive neurons is representative of mean values ± SEM from three independent biological replicates. More than 95% of RA-treated SHSY-5Y cells established a cholinergic-like phenotype by displaying ChAT-like immunoreactivity at culture day three.

The sequential treatment of exponentially growing SH-SY5Y cells with all-trans RA induced a distinctive morphology resembling that of cholinergic neurons [32]. Following three consecutive days, immunocytochemical evaluations of cholinergic neuronal marker ChAT revealed that more than 95% of RA-differentiated SH-SY5Y cells robustly expressed ChAT (*** *p* < 0.001) (Figure 1).

### 2.2. Low-Dose Exposure to KP-10 Has No Effect on RA-Differentiated Cholinergic Cell Viability

**Figure 2:**
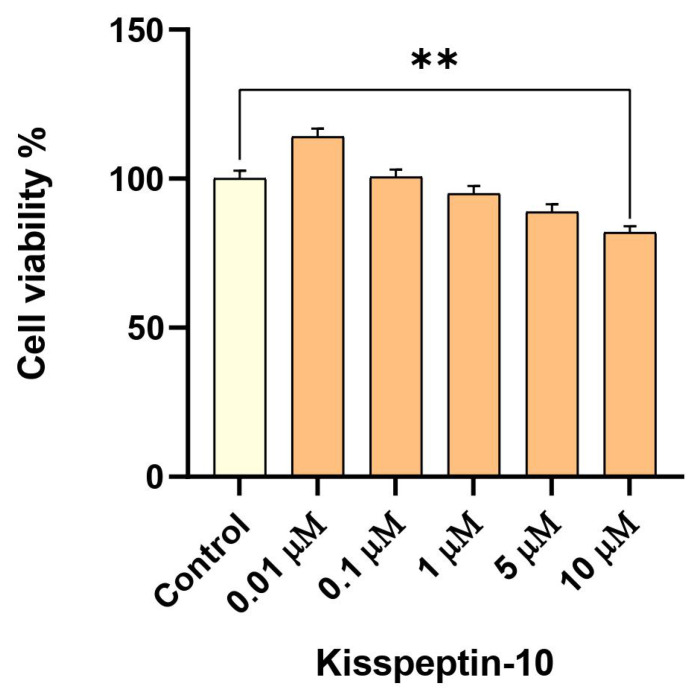
The effect of kisspeptin-10 (KP-10) on retinoic acid (RA)-differentiated cholinergic cell viability. Cholinergic differentiated SH-SY5Y cells were exposed to KP-10 in the concentration range between 0.01 to 10 µM for 24 h. The data presented in Figure 2 are representative of mean values ± SEM from three independent biological replicates performed in triplicates. While low doses of KP-10 (0.01–5 µM) displayed no significant differences in cellular viability, 10 µM of KP-10 decreased cell survival down to 80%. Statistical significance was defined as *p* value less than 0.01 (** *p* < 0.01).

To verify that KP-10 by itself has no modulatory effect on cellular proliferation or cytotoxicity that may obscure its neuroprotective potential against α-syn-induced toxicity, differentiated SHSY-5Y neurons were first exposed to various doses of KP-10 and assayed for 3-(4, 5-dimethylthiazole-2-yl)-2, 5-dipenyltetrazolium bromide (MTT) uptake. Analysis of cell viability following a 24-h treatment showed that KP-10 in the concentration range between 0.01 to 5 µM has a negligible influence on RA-differentiated cholinergic cells. By contrast, treatment of cultured cells with 10 µM of KP-10 significantly reduced cell survival down to 80% after 24 h (** *p* < 0.01) (Figure 2). 

### 2.3. α-Syn mRNA Overexpression in RA-Differentiated Cholinergic Cells

**Figure 3:**
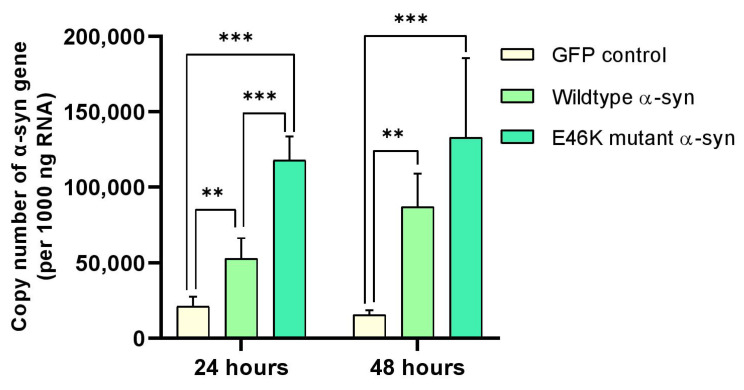
α-Synuclein (α-syn) mRNA overexpression in retinoic acid (RA)-differentiated cholinergic cells. Cholinergic differentiated cells were transfected with human wild-type or E46K mutant α-syn for 24 h and 48 h. The data presented in Figure 3 are representative of mean values ± SEM from three independent biological replicates performed in duplicates. One-way analysis of variance (ANOVA) followed by Tukey’s post hoc test indicated considerable increments of α-syn copy number in cells overexpressing human wild-type (** *p* < 0.01) and E46K mutant α-syn (*** *p* < 0.001) 24 h post-transfection compared with the green fluorescence protein (GFP)-expressing cells. Similar changes of α-syn mRNA expression were observed in cholinergic differentiated cells 48 h post-transfection compared with the GFP control (wild-type α-syn, ** *p* < 0.01; E46K mutant α-syn, *** *p* < 0.001). Statistical significance was defined as *p* value less than 0.01 and 0.001 (** *p* < 0.01 and *** *p* < 0.001).

To validate the overexpression of α-syn mRNA, we utilized the conventional approach to transiently transfect α-syn expression vectors into RA-differentiated cholinergic cells and consecutively analyzed the upregulation of α-syn mRNA expression by quantitative real-time PCR (qPCR) at 24 h and 48 h post-transfection. Accordingly, significant increases of α-syn copy number in cells overexpressing human wild-type (** *p* < 0.01) and E46K mutant α-syn (*** *p* < 0.001) were observed 24 h post-transfection compared with the green fluorescence protein (GFP)-expressing cells. Comparable alterations of α-syn mRNA levels were likewise discerned in cholinergic differentiated cells 48 h post-transfection compared with the GFP control (wild-type α-syn, ** *p* < 0.01; E46K mutant α-syn, *** *p* < 0.001) (Figure 3).

### 2.4. Elevated α-Syn mRNA Levels Exacerbate Cellular Toxicity in RA-Differentiated Cholinergic Cells

**Figure 4:**
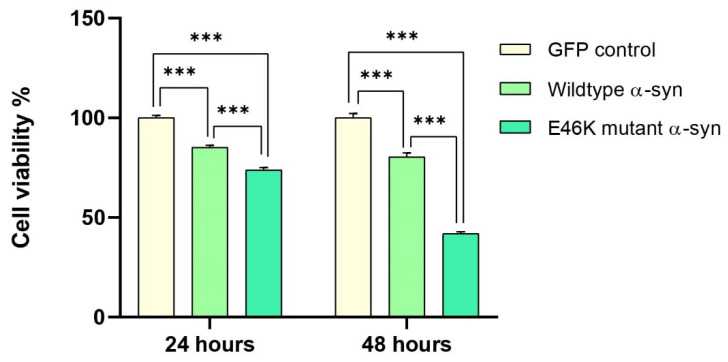
The functional effects of human wild-type or E46K mutant α-synuclein (α-syn) overexpression on retinoic acid (RA)-differentiated cholinergic cell viability. Cholinergic differentiated SH-SY5Y cells were transiently transfected with human wild-type or E46K mutant α-syn for 24 h and 48 h before being subjected to 3-(4, 5-dimethylthiazole-2-yl)-2, 5-dipenyltetrazolium bromide (MTT) analysis for cellular viability. The data presented in Figure 4 are representative of mean values ± SEM from three independent biological replicates performed in triplicates. While cells overexpressing human wild-type α-syn displayed ~85% survival at 24 h (*** *p* < 0.001) and ~80% survival at 48 h (*** *p* < 0.001) post-transfection, cells overexpressing E46K mutant α-syn exhibited ~74% survival at 24 h (*** *p* < 0.001) and ~40% survival at 48 h (*** *p* < 0.001) post-transfection. Statistical significance was defined as *p* value less than 0.001 (*** *p* < 0.001).

The functional consequences of human wild-type or E46K mutant α-syn overexpression on RA-differentiated cholinergic cell viability were subsequently evaluated by the MTT-based cytotoxicity assay. Notably, cholinergic differentiated cells overexpressing human wild-type α-syn displayed ~85% survival at 24 h (*** *p* < 0.001) and ~80% survival at 48 h (*** *p* < 0.001) post-transfection. There was, however, a lower survival rate in cultured cells overexpressing E46K mutant α-syn, which unveiled ~74% survival at 24 h (*** *p* < 0.001) and ~40% survival at 48 h (*** *p* < 0.001) post-transfection (Figure 4).

### 2.5. KP-10 Mitigates α-Syn-Induced Toxicity in RA-Differentiated Cholinergic Cells

**Figure 5:**
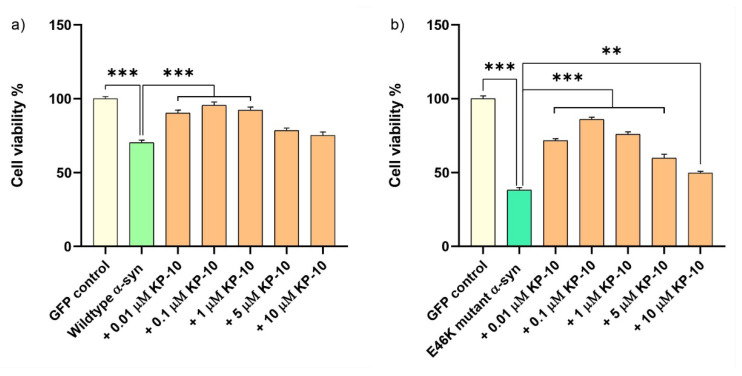
The effect of kisspeptin-10 (KP-10) on human wild-type or E46K mutant α-synuclein (α-syn)-induced toxicity in retinoic acid (RA)-differentiated cholinergic cells. Differentiated cells overexpressing human wild-type or E46K mutant α-syn were subjected to 24 h of exogenous KP-10 (0.01–10 µM) treatment. The data presented in Figure 5 are representative of mean values ± SEM from three independent biological replicates performed in triplicates. While wild-type α-syn-induced toxicity was considerably inhibited by 0.01, 0.1 and 1 μM of KP-10 (*** *p* < 0.001) (**a**), E46K mutant α-syn-induced toxicity was markedly suppressed by 0.01, 0.1, 1, 5 (*** *p* < 0.001) and 10 μM (** *p* < 0.01) of KP-10 (**b**). Statistical significance was defined as *p* value less than 0.01 and 0.001 (** *p* < 0.01 and *** *p* < 0.001).

In order to delineate the neuroprotective efficacy of KP-10 against α-syn-induced toxicity, cholinergic differentiated SHSY-5Y cells overexpressing human wild-type or E46K mutant α-syn were subjected to 24 h of exogenous KP-10 (0.01–10 µM) treatment, following which cellular viability was assessed. Remarkably, while wild-type α-syn-induced toxicity was significantly inhibited by 0.01, 0.1 and 1 µM of KP-10 (*** *p* < 0.001) (Figure 5a), E46K mutant α-syn-induced toxicity was substantially suppressed by 0.01, 0.1, 1, 5 (*** *p* < 0.001) and 10 µM (** *p* < 0.01) of KP-10 (Figure 5b). 

### 2.6. KP-10 Binds to the C-Terminal Residues of Wild-Type and E46K Mutant α-Syn In Silico

**Figure 6:**
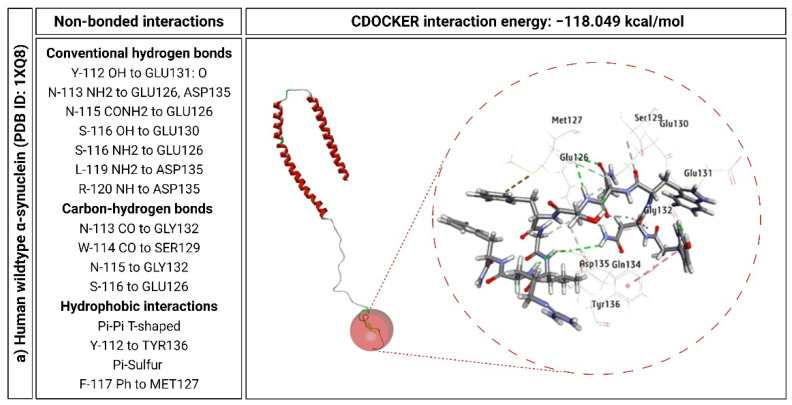

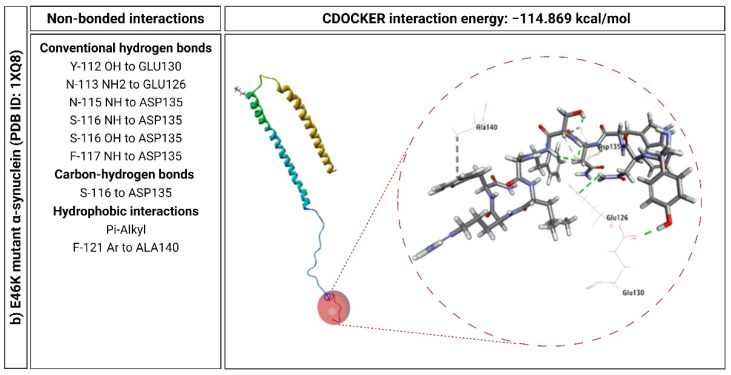
Computational models of human wild-type (PDB ID: 1XQ8) and E46K mutant α-synuclein (α-syn) (PDB ID: 1XQ8) depicting specific binding sites and intermolecular interactions with kisspeptin-10 (KP-10). Key amino acid residues of KP-10 (112–117) formed hydrogen bonds (blue and white) and hydrophobic interactions (magenta) with human wild-type (**a**) and E46K mutant α-syn (**b**). The lysine residue is shown in the E46K mutant α-syn.

**Figure 7:**
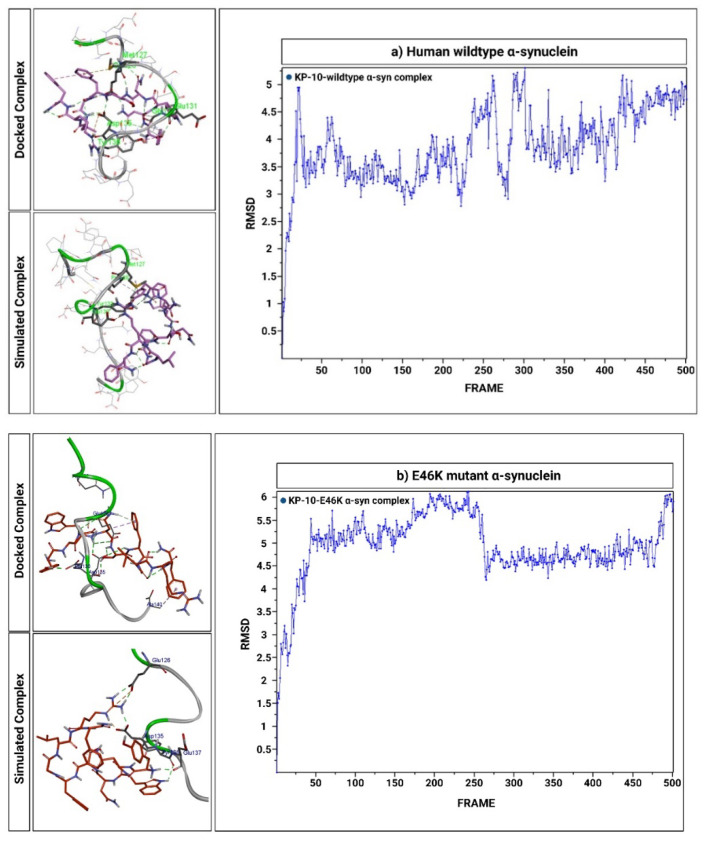
Docked (before molecular dynamics) and simulated (after molecular dynamics) complexes of kisspeptin-10 (KP-10)-wild-type α-synuclein (α-syn) (**a**) and KP-10-E46K mutant α-syn (**b**), along with their respective root-mean-square deviation (RMSD) values plotted as a function of trajectory frame. Despite α-syn’s inherent conformational flexibility, bound KP-10 molecules stabilized after 200 trajectory frames.

The intermolecular binding interactions between KP-10 and human wild-type or E46K mutant α-syn were initially probed recursively based upon CDOCKER interaction energies (-kcal/mol). Relying on binding site topology and intermolecular affinity, a lower CDOCKER interaction energy implies greater binding affinity. According to this parameter, KP-10 binds favorably to wild-type α-syn with a CDOCKER energy score of −118.049 kcal/mol. Detailed evaluation of docked poses further revealed that key amino acid residues of KP-10 (112–117) generated hydrogen bonds with Glu126, Glu130, Glu131, Gly132, and Asp135 of wild-type α-syn. Intriguingly, the binding mode assessment of KP-10 with E46K mutant α-syn displayed analogous binding patterns to the same binding site, generating hydrogen bonds with Glu126, Glu130, and Asp135 of E46K mutant with a docking score of −114.869 kcal/mol. The docked conformations of KP-10 with human wild-type or E46K mutant α-syn, along with key interactions between putative binding site residues, are illustrated in Figure 6a,b. In this context, although in silico predictions rationalized the surface complementarities between KP-10 and both α-syns in terms of docking affinities, biological constraints such as protein flexibility and solvation dynamics were not fully considered. Thus, to compensate for the aforementioned limitations, MD simulations were consecutively executed to comprehend the time-dependent stability of KP-10-α-syn complexes in an explicit solvent environment. Based on the simulation’s trajectories, the conformational landscape of docked complexes was subsequently parameterized according to root-mean-square deviation (RMSD) values. Herein, we report that KP-10 was unambiguously accommodated spatially in the C-terminal binding pockets of both α-syns throughout the course of the simulation. While the wild-type and E46K mutant α-syn mechanically unfolded into relatively linear configurations, with their respective conformational flexibilities increasing monotonically towards the very C-terminus, bound KP-10 molecules stabilized after 200 trajectory frames. The MD trajectories of docked KP-10-α-syn complexes, along with their corresponding RMSD values, are depicted in Figure 7a,b and Supplementary Videos S1 and S2.

## 3. Discussion

ChAT-positive SH-SY5Y neurons were first engineered ad hoc to transiently overexpress human wild-type or E46K mutant α-syn, while α-syn-mediated cytotoxicity was subsequently quantified using an MTT-based metabolic assay that determines neuronal survival. Cumulatively, overexpression of the pathological mutation E46K exhibited elevated α-syn mRNA levels, which correlated more distinctly with the degree of cellular toxicity as compared to its wild-type counterpart in cholinergic neuronal-like cells. Our results, thereby, cohere with the hypothesis that a pronounced upsurge of α-syn mRNA expression over baseline levels could have detrimental consequences against cholinergic neurons, efficiently mimicking the neuropathology of DLB. The up-regulation of human wild-type and E46K mutant α-syn as a result of aberrant gene dosage has consistently augmented the formation of extracellular aggregation-prone morphologies that yielded neuronal toxicity when overexpressed in cellular models [33,34,35]. Given that α-syn-laden cholinergic neurons are a prerequisite for exacerbated DLB pathology [36,37,38], various approaches have been employed to abrogate the toxic insults of aggregated α-syn by counteracting extra- and intracellular pathogenic pathways [39,40,41]. Such approaches range from the extracellular enhancement of chaperone-based therapeutics that refold aberrant α-syn conformations [16] to strategies aimed at inhibiting intracellular beta-pleated α-syn aggregates [39,42]. As such, since the NAC fragment of human α-syn binds preferentially to Aβ and facilitates its abnormal aggregation [10], it is tempting to speculate that compounds which specifically bind to extracellular α-syn or Aβ may be neuroprotective [22,43]. To address this multi-hit hypothesis, we then explored whether exogenous administration of KP-10 might play a critical role in the attenuation of neuronal cell death induced by α-syn in ChAT-positive neurons. Upon treatment with exogenous KP-10, while a high concentration of KP-10 (10 µM) unexpectedly decreased the cellular viability of RA-differentiated cholinergic cells, low concentrations of KP-10 (0.01–1 µM) significantly suppressed wild-type and E46K mutant α-syn-induced toxicity. In marked contrast, however, an altered dose-response curve was observed in cholinergic differentiated cells following low dose exposure to KP-10 (0.01–0.1 µM), with an apparent decrease in neuroprotective efficacy when in the presence of wild-type or E46K mutant α-syn. This discrepancy in response variability is indicative of a possibility that α-syn may be compromising the intracellular transduction of kisspeptin-GPR54 signaling in RA-differentiated cholinergic cells. More sophisticated receptor binding studies may shed light on this possibility, but such a phenomenon might only be demonstrable under dynamic conditions in which cellular responses are investigated via a GPR54 antagonist. Consistent with the in vitro outcomes are theoretical predictions from molecular docking algorithms, which demonstrated that KP-10 binds favorably to specific sites of wild-type and E46K mutant α-syn with CDOCKER interaction energies of −118.049 kcal/mol and −114.869 kcal/mol, respectively. In particular, key amino acid residues of KP-10 (112–117) were found to have generated hydrogen bonds with the C-terminal residues (Glu126, Glu130, Glu131, Gly132, and Asp135) of α-syn. A visual inspection of MD trajectories over a 50 ns time-scale further suggested that the KP-10-α-syn complexes were relatively stable despite solvent-driven perturbations. Throughout the span of the simulation, KP-10 re-docked into the C-terminus of both α-syns upon disassembly, thus reflecting the existence of a putative binding site. Although the mechanistic details of these “stable adducts” are somewhat arbitrary, the elucidation of these findings may insinuate otherwise. It became apparent from early in vitro experimentations that transient long-range tertiary interactions between the N and C termini of α-syn are crucial for spontaneous NAC-mediated aggregation [44,45,46,47,48]. Contrary to expectations, naturally existing compounds that bind to α-syn with binding sites at both N- and C-termini have been shown to redirect aggregation-prone α-syn into off-pathway non-toxic assemblies [49,50,51,52]. Intriguingly, the α-syn folds back onto itself to form a loop that inhibits α-syn aggregation since the NAC fragment is no longer primed to create a linear conformation required for the formation of β-sheet-rich aggregates [44,53]. Guided by this notion, natural molecules that bind to the C-terminal residues of α-syn were able to shield the C-terminus from bimolecular self-assembly and preclude intramolecular contacts between the N and C termini, thereby impeding misfolding and ensuing aggregation [54,55,56,57]. Taking these observations into consideration, it seems plausible that the 6-residue fragment (112–117) of KP-10 could be specifically binding to the C-terminus of α-syn by mediating a physical influence that attenuates the likelihood of misfolding to confer neuroprotection in vitro. The fact that the exact peptide fragment was able to bind predominantly to extracellular Aβs to promote neuronal survival [22] provides credence to the notion that KP-10’s binding zone may harness functional moieties of neuroprotective significance. We thus conclude from these complementary investigations that the neuroprotective relevance of KP-10 binding mechanisms can be structurally and functionally implicated in α-syn-mediated toxicity of cholinergic neuronal-like cells. Stemming from this purported facet, further exploration into KP-10’s mode of action may provide the basis for novel structure-based drug design of α-syn-interfering aggregation inhibitors. Indeed, the identification of this non-canonical binding interface endows KP-10 with the ability to engender a previously unattainable level of pharmacological selectivity by targeting not just the α-syn but specific pro-survival signaling cascades. However, as it probably represents a biologically relevant interaction with the kisspeptin-GPR54 signaling pathway, subsequent biophysical characterizations by virtue of GPR54 antagonism are warranted.

## 4. Materials and Methods

### 4.1. KP-10

KP-10 (amino acids 112–121), obtained as 1 mg of lyophilized powder (Tocris Bioscience, Bristol, UK; Cat. #: 2570), was dissolved in Milli-Q water according to the manufacturer’s recommendations to prepare a stock solution of 500 µM that was further diluted with distilled water (dH2O).

### 4.2. α-Syn Plasmids

The pcDNA6 human wild-type (Insert size: 906 bp) and E46K mutant α-syn (Insert size: 1073 bp) plasmids were kindly provided by Prof. Hilal Lashuel (Addgene, Watertown, MA, USA; plasmid nos. 107425 and 105730) [58], while the enhanced green fluorescence protein (pEGFP-C1) control plasmid was obtained from Clontech Laboratories (BD Biosciences, Palo Alto, CA, USA; plasmid no. 6084-1). The DNA sequence of cloned inserts was next subjected to Sanger sequencing for confirmation at Apical Scientific Sdn. Bhd. (Selangor, Malaysia). All plasmid constructs were eventually purified with a Wizard Plus SV Minipreps DNA Purification System (Promega, Madison, WI, USA; Cat. #: A1330). 

### 4.3. Cell Culture

The human neuroblastoma SH-SY5Y cell line purchased from ATCC (Manassas, VA, USA; Cat. #: CRL-2266) was cultivated in 25 cm^2^ cell culture flasks at 37 °C under a humidified atmosphere of 5% CO^2^ in Dulbecco’s modified Eagle medium (DMEM) with high glucose and L-glutamine (Nacalai Tesque, Kyoto, Japan; Cat. #: 08469-35), supplemented with 10% fetal bovine serum (FBS) (Thermo Fisher Scientific, Waltham, MA, USA; Cat. #: 10100147) and 1% penicillin-streptomycin (MilliporeSigma, Burlington, MA, USA; Cat. #: P0781). The medium was replenished every three days, and at a confluence of approximately 80%, cells were detached with Accutase (Nacalai Tesque, Kyoto, Japan; Cat. #: 12679-54), sub-cultured into 25 cm^2^ culture flasks, and plated for assays.

### 4.4. Differentiation of SHSY-5Y into Cholinergic Neurons

Cholinergic neuronal-like cells were generated using a differentiation procedure modified from a previously described protocol [30]. Briefly, SHSY-5Y cells were plated in DMEM containing 0.5% FBS and 10 μM of all-trans retinoic acid (RA) (Sigma–Aldrich, St Louis, MO, USA; Cat. #: R2625) for 72 h to induce cholinergic differentiation. After three days in the presence of all-trans RA, the differentiation medium was replenished with DMEM containing 10% FBS for the transient transfection of plasmids. In general, cells were used between passages 17 to 19 and were never passaged beyond passage 19 in order to avoid any potential phenotypic change in RA-differentiated cholinergic cells.

### 4.5. Plasmid Transfections

The transient transfection of plasmids in RA-differentiated cholinergic cells was accomplished by utilizing Lipofectamine 3000 reagents (Thermo Fisher Scientific, Waltham, MA, USA; Cat. #: L3000001) to ensure maximal gene expression with minimal cellular toxicity. Briefly, 0.1 μg of pEGFP-C1, 0.1 μg of pcDNA6 wild-type α-syn, or 0.1 μg of pcDNA6 E46K mutant α-syn plasmids were diluted in 5 μL of Opti-MEM I reduced serum medium (Thermo Fisher Scientific, Waltham, MA, USA; Cat. #: 31985070) and 0.2 μL of P3000 reagent. Following incubation for 5 min at room temperature (RT), 0.3 µL of Lipofectamine 3000 reagent was diluted in 5 μL of Opti-MEM I reduced serum medium, added into the above-diluted Opti-MEM-DNA solution, and incubated for an additional 15 min to form DNA-Lipofectamine 3000 complexes. The complex mixtures were then added dropwise into each well of a 96-well plate and incubated in a humidified 5% CO^2^ incubator for two to four days. Finally, the evaluation of transfection efficiency by visualization of GFP expression in RA-differentiated cholinergic cells was done by counting the number of GFP-positive cells using a Neubauer hemocytometer under a Nikon Eclipse 90i fluorescent microscope (Nikon, Melville, NY, USA). 

### 4.6. Immunofluorescent Staining

RA-differentiated cholinergic cells were first fixed with 4% paraformaldehyde in phosphate-buffered saline (PBS) (pH 7.4) for 20 min at RT. Prior to immunostaining, differentiated cells were blocked and permeabilized with PBS containing 10% normal goat serum (NGS), 1% bovine serum albumin (BSA), and 0.3% Triton X-100 for 45 min. Subsequently, primary antibodies were applied to the cells and incubated overnight at 4 °C. The primary antibodies used were goat polyclonal anti-ChAT (1:1000; MilliporeSigma, Burlington, MA, USA; Cat. #: AB144P). After three washing steps with PBS, cells were incubated with secondary antibodies, donkey anti-goat Alexa Fluor 488 (1:200; Thermo Fisher Scientific, Waltham, MA, USA; Cat. #: A-11055) for 1 h at RT. Cell nuclei were next stained with 4′, 6-diamidino-2-phenylindole (DAPI) (1:1000; Thermo Fisher Scientific, Waltham, MA, USA; Cat. #: 62247) for 1 min. Differentiated cells were finally visualized and enumerated using a Nikon 90i fluorescent microscope equipped with a DXM1200C Nikon digital camera (Nikon, Melville, NY, USA). Upon treatment with RA for three days, SH-SY5Y cells developed a predominantly cholinergic-like phenotype with homogenous generations of ChAT-immunoreactive neurons. Since ChAT is an acetylcholine-synthesizing transferase, its presence classifies a nerve cell as a cholinergic neuron. 

### 4.7. Cell Viability Assay

SHSY-5Y cells were seeded in technical triplicates into 96-well plates (NEST Biotechnology, Wuxi, China; Cat. #: 701001) at a density of 3 × 10^4^ cells/well and incubated for a period of 24 h. Following RA-induced differentiation and transient transfection of plasmids, 20 μL of KP-10 peptides were added to each well and incubated for an additional 24 h in 5% CO^2^ at 37 °C. To determine the appropriate dose for neuroprotection, differentiated transfected cells were cultured with various concentrations (0.01–10 µM) of KP-10. Subsequently, 20 μL of MTT reagent (Thermo Fisher Scientific, Waltham, MA, USA; Cat. #: M-6494) was added to a final concentration of 0.5 mg/mL for 4 h of incubation at 37 °C. Finally, the MTT-containing medium was carefully siphoned out, and 150 μL of dimethyl sulfoxide (DMSO) (Sigma–Aldrich, St Louis, MO, USA; Cat. #: D8418) was added to each well to solubilize the formazan crystals that had formed. The absorbance of each well was measured at 570 nm with a reference wavelength of 650 nm on a microplate reader (TECAN Infinite M200 Pro, Männedorf, Switzerland). The background light scattering at 650 nm was then subtracted from the values obtained for formazan absorbance (570 nm).

### 4.8. RNA Isolation and cDNA Synthesis

The RNA isolation of α-syn-transfected RA-differentiated cholinergic cells was performed by first dissolving the cells in 200 µL of Tri-RNA reagent (Sigma–Aldrich, St Louis, MO, USA; Cat. #: T9424). Chloroform was then added in a 1:5 ratio and shaken to mix thoroughly, followed by centrifugation at 12,000 g, 4 °C for 15 min. The aqueous phases were carefully removed into fresh centrifuge tubes, and isopropyl alcohol was then added at a 1:2 ratio. The mixtures were next incubated at RT for 10 min, followed by centrifugation at 12,000*×* *g*, 4 °C for 15 min. The supernatants produced were consequently discarded while the pellets were rinsed with 75% ethanol, followed by centrifugation at 7500 g, 4 °C for 5 min. The pellets were then air-dried for 5 min and subsequently dissolved in 30 µL of Milli-Q water. The purity of the RNAs extracted was determined using the nanodrop-1000 spectrophotometer (Thermo Fisher Scientific, Waltham, MA, USA). RNA isolations (initial input amounts of 1000 ng) with a 260/280 value of above 1.90 were deemed as pure and reverse transcribed into cDNAs using the High Capacity cDNA Reverse Transcription Kit (Applied Biosystems, Foster City, CA, USA; Cat. #: 4374966).

### 4.9. Quantification of α-Syn mRNA Using qPCR

The absolute copy number of the human *α-syn* gene was determined using primer sequences listed as follows: human α-syn: Forward primer, 5′-TATCTGTACCTGCCCCCACT-3′ and Reverse primer, 5′-GCCACAAAATCCACAGCACA-3′ (PCR product length: 95 base pairs, GenBank accession number: NM_001146055.1); human inosine 5’-monophosphate dehydrogenase 2 (IMPDH2): Forward primer, 5′-GCAATGGCGCTTACAGGC-3′ and Reverse primer, 5′-GGGTCTGTGATGAATCCCTGT-3′ (PCR product length: 113 base pairs, GenBank accession number: NM_000884.3). Briefly, PCR products produced by the human α-syn primers were ligated into pGEM-T Easy Vectors (Promega, Madison, WI, USA; Cat. #: A1360), transformed into competent DH5α Escherichia coli cells (Thermo Fisher Scientific, Waltham, MA, USA; Cat. #: EC0112), and subsequently harvested using the Wizard Plus SV Minipreps DNA Purification System (Promega, Madison, WI, USA). The cloned vectors were then subjected to Sanger sequencing for confirmation at Apical Scientific Sdn. Bhd. (Selangor, Malaysia). Next, plasmids containing the α-syn gene were serially diluted to concentrations of 10^9^, 10^8^, 10^7^, 10^6^, 10^5^, 10^4^, 10^3^, and 10^2^ copy/μL and used as standards for the absolute quantification of α-syn mRNA. The Sensi FAST SYBR Hi-ROX Kit (Bioline, Cincinnati, Ohio, USA; Cat. #: BIO-92005) was utilized to perform qPCR with the cycling conditions as follows: 95 °C for 2 min, 95 °C for 15 s, 40 cycles of 60 °C for 30 s, and a final dissociation step for melting curve analysis. The absolute copy number of α-syn mRNAs was eventually determined according to the generated standard curve. All data were normalized to the housekeeping gene human IMPDH2 and analyzed with the 7500 Real-Time PCR Software (Applied Biosystems, Foster City, CA, USA).

### 4.10. In Silico Molecular Docking

The X-ray crystal structure of human α-syn was first retrieved from the Protein Data Bank (www.rcsb.org; Accessed on 21 May 2021) with a PDB ID of 1XQ8. KP-10 was next constructed in ChemDraw Professional 15.0 with the peptide sequence of H-YNWNSFGLRF-[NH2], identical to the ones used in vitro. All computational-based analyses were eventually carried out using the docking software CDOCKER with BIOVIA Discovery Studio 4.0 (San Diego, CA, USA). CDOCKER is an algorithm that employs CHARMm force fields to adopt a basic strategy involving the generation of several initial ligand orientations in the active site of target proteins followed by molecular dynamics-based simulated annealing and a final refinement via energy minimization [59]. Accordingly, α-syn and KP-10 proteins were energy-minimized using the CHARMm force field. The binding site of α-syn was then retrieved from the ‘receptor cavities’ of the software and utilized for docking purposes. KP-10 was consequently docked into the probable binding site of α-syn to obtain the best possible conformations, which are built upon total docking energy. The pose or conformation having the highest dock score was deemed as having the most favorable interaction. This procedure was first performed on the wild-type α-syn and subsequently repeated for the E46K mutant. For the virtual mutation of wild-type α-syn, the ‘Build Mutant’ protocol was used for the substitution of amino acid residues. Here, using wild-type α-syn as a template, the E46K mutant was generated by substituting glutamic acid with lysine on the protein sequence. Energy minimization for the optimization of residue geometry was then performed on the E46K mutant using the algorithm of smart minimization until gradient tolerance (RMS Gradient 0.1 kcal/mol/A°) was satisfied. KP-10 was ultimately docked into the same binding site as in the case of the wild-type α-syn. 

### 4.11. Explicit-Solvent MD Simulations

To parameterize the dynamic range of KP-10-α-syn complexes in an explicit solvent, MD simulations were carried out within the YASARA graphical user interface using the Amber 14 force field [60]. Briefly, all MD simulations utilized an explicit solvation system described by the TIP3P water model and a cubic periodic boundary that extended 20 Å around the KP-10-α-syn complexes. Electrostatic interactions were predicated by the Particle mesh Ewald (PME) method for long-range coulombic forces. The entire system was energy-minimized with 5000 steps of steepest descent followed by 5000 steps of conjugate gradients to eradicate conformational stress. In order to mimic physiological conditions, KP-10-α-syn interactions were solvated with water molecules and subsequently neutralized with counter ions with 0.9% NaCl salt at 303 K temperature. The temperature of the simulation system was governed by the Berendsen thermostat, and the final production phase of simulations was successively carried out for a total of 50 ns MD simulations. Finally, the trajectory frames were calculated with a time step of 1.25 fs, and the simulation’s snapshots were captured every 100 ps. MD simulations for both wild-type and E46K mutant α-syn complexes were probed under identical experimental conditions. 

### 4.12. Statistical Analysis

Data were presented as mean ± SE from three independent biological experiments. Statistical analysis was evaluated using one-way analysis of variance (ANOVA) followed by Tukey’s post hoc tests for all multiple comparisons (IBM SPSS Statistics v24). A *p*-value of less than 0.05 was defined as a statistically significant difference.

## Data Availability

The data presented in this study are available within the article. The raw data supporting the conclusions of this manuscript will be made available by the authors, without undue reservation, to any qualified researcher.

## Supplementary Materials

The following supporting information can be downloaded at: https://www.mdpi.com/article/10.3390/ijms23095193/s1.

## References

[1] Pepeu G., Giovannini M.G. (2017). The fate of the brain cholinergic neurons in neurodegenerative diseases. Brain Res..

[2] Gómez-Tortosa E., Newell K., Irizarry M.C., Sanders J.L., Hyman B.T. (2000). α-Synuclein immunoreactivity in dementia with Lewy bodies: Morphological staging and comparison with ubiquitin immunostaining. Acta Neuropathol..

[3] Simon C., Soga T., Okano H.J., Parhar I. (2021). α-Synuclein-mediated neurodegeneration in Dementia with Lewy bodies: The pathobiology of a paradox. Cell Biosci..

[4] Zarranz J.J., Alegre J., Gomez-Esteban J.C., Lezcano E., Ros R., Ampuero I., Vidal L., Hoenicka J., Rodriguez O., Atarés B. (2004). The new mutation, E46K, of α-synuclein causes parkinson and Lewy body dementia. Ann. Neurol..

[5] Colom-Cadena M., Gelpi E., Charif S., Belbin O., Blesa R., Martí M.J., Clarimón J., Lleó A. (2013). Confluence of α-Synuclein, Tau, and β-Amyloid Pathologies in Dementia With Lewy Bodies. J. Neuropathol. Exp. Neurol..

[6] Swirski M., Miners J.S., De Silva R., Lashley T., Ling H., Holton J., Revesz T., Love S. (2014). Evaluating the relationship between amyloid-β and α-synuclein phosphorylated at Ser129 in dementia with Lewy bodies and Parkinson’s disease. Alzheimer’s Res. Ther..

[7] Lashuel H.A., Overk C.R., Oueslati A., Masliah E. (2013). The many faces of α-synuclein: From structure and toxicity to therapeutic target. Nat. Rev. Neurosci..

[8] Han H., Weinreb P.H., Lansbury Jr P.T. (1995). The core Alzheimer’s peptide NAC forms amyloid fibrils which seed and are seeded by β-amyloid: Is NAC a common trigger or target in neurodegenerative disease?. Chem. Biol..

[9] Jensen P.H., Sørensen E.S., Petersen T.E., Gliemann J., Rasmussen L.K. (1995). Residues in the synuclein consensus motif of the *α*-synuclein fragment, NAC, participate in transglutaminase-catalysed cross-linking to Alzheimer-disease amyloid *β*A4 peptide. Biochem. J..

[10] Jensen P.H., Højrup P., Hager H., Nielsen M.S., Jacobsen L., Olesen O.F., Gliemann J., Jakes R. (1997). Binding of A*β* to α- and *β*-synucleins: Identification of segments in α-synuclein/NAC precursor that bind A*β* and NAC. Biochem. J..

[11] Lloyd G.M., Dhillon J.-K.S., Gorion K.-M.M., Riffe C., Fromholt S.E., Xia Y., Giasson B.I., Borchelt D.R. (2021). Collusion of α-synuclein and Aβ aggravating co-morbidities in a novel prion-type mouse model. Mol. Neurodegener..

[12] Stuendl A., Kunadt M., Kruse N., Bartels C., Moebius W., Danzer K.M., Mollenhauer B., Schneider A. (2016). Induction of α-synuclein aggregate formation by CSF exosomes from patients with Parkinson’s disease and dementia with Lewy bodies. Brain.

[13] Ngolab J., Trinh I., Rockenstein E., Mante M., Florio J., Trejo M., Masliah D., Adame A., Masliah E., Rissman R.A. (2017). Brain-derived exosomes from dementia with Lewy bodies propagate α-synuclein pathology. Acta Neuropathol. Commun..

[14] Ottolini D., Calì T., Szabo I., Brini M. (2017). Alpha-synuclein at the intracellular and the extracellular side: Functional and dysfunctional implications. Biol. Chem..

[15] Clinton L.K., Blurton-Jones M., Myczek K., Trojanowski J.Q., LaFerla F.M. (2010). Synergistic Interactions between Aβ, Tau, and -Synuclein: Acceleration of Neuropathology and Cognitive Decline. J. Neurosci..

[16] Lee H.-J., Bae E.-J., Lee S.-J. (2014). Extracellular α-synuclein—a novel and crucial factor in Lewy body diseases. Nat. Rev. Neurol..

[17] Bassil F., Brown H.J., Pattabhiraman S., Iwasyk J.E., Maghames C.M., Meymand E.S., Cox T.O., Riddle D.M., Zhang B., Trojanowski J.Q. (2020). Amyloid-Beta (Aβ) Plaques Promote Seeding and Spreading of Alpha-Synuclein and Tau in a Mouse Model of Lewy Body Disorders with Aβ Pathology. Neuron.

[18] Mills E.G., O’Byrne K.T., Comninos A.N. (2019). Kisspeptin as a Behavioral Hormone. Semin. Reprod. Med..

[19] Rumpler E., Skrapits K., Takács S., Göcz B., Trinh S.H., Rácz G., Matolcsy A., Kozma Z., Ciofi P., Dhillo W.S. (2021). Characterization of kisspeptin neurons in the human rostral hypothalamus. Neuroendocrinology.

[20] Ibos K.E., Bodnár E., Bagosi Z., Bozsó Z., Tóth G., Szabó G., Csabafi K. (2021). Kisspeptin-8 Induces Anxiety-Like Behavior and Hypolocomotion by Activating the HPA Axis and Increasing GABA Release in the Nucleus Accumbens in Rats. Biomedicines.

[21] Tanaka M., Csabafi K., Telegdy G. (2013). Neurotransmissions of antidepressant-like effects of kisspeptin-13. Regul. Pept..

[22] Milton N.G.N., Chilumuri A., Rocha-Ferreira E., Nercessian A.N., Ashioti M. (2012). Kisspeptin Prevention of Amyloid-β Peptide Neurotoxicity in Vitro. ACS Chem. Neurosci..

[23] Chilumuri A., Ashioti M., Nercessian A.N., Milton N.G.N. (2013). Immunolocalization of Kisspeptin Associated with Amyloid-β Deposits in the Pons of an Alzheimer’s Disease Patient. J. Neurodegener. Dis..

[24] Messager S., Chatzidaki E.E., Ma D., Hendrick A.G., Zahn D., Dixon J., Thresher R.R., Malinge I., Lomet D., Carlton M.B.L. (2005). Kisspeptin directly stimulates gonadotropin-releasing hormone release via G protein-coupled receptor 54. Proc. Natl. Acad. Sci. USA.

[25] Chilumuri A., Milton N.G.N. (2013). The Role of Neurotransmitters in Protection against Amyloid-β Toxicity by KiSS-1 Overexpression in SH-SY5Y Neurons. ISRN Neurosci..

[26] Tagliafierro L., Chiba-Falek O. (2016). Up-regulation of SNCA gene expression: Implications to synucleinopathies. Neurogenetics.

[27] Neystat M., Lynch T., Przedborski S., Kholodilov N., Rzhetskaya M., Burke R.E. (1999). α-Synuclein expression in substantia nigra and cortex in Parkinson’s disease. Mov. Disord..

[28] Taylor J.-P., Collerton D., LeBeau F., Perry E. (2017). Cholinergic Pathology in Dementia with Lewy Bodies. Dementia with Lewy Bodies.

[29] Xicoy H., Wieringa B., Martens G.J.M. (2017). The SH-SY5Y cell line in Parkinson’s disease research: A systematic review. Mol. Neurodegener..

[30] de Medeiros L.M., De Bastiani M.A., Rico E.P., Schonhofen P., Pfaffenseller B., Wollenhaupt-Aguiar B., Grun L., Barbé-Tuana F., Zimmer E.R., Castro M.A.A. (2019). Cholinergic Differentiation of Human Neuroblastoma SH-SY5Y Cell Line and Its Potential Use as an In vitro Model for Alzheimer’s Disease Studies. Mol. Neurobiol..

[31] Korecka J.A., van Kesteren R., Blaas E., Spitzer S.O., Kamstra J., Smit A.B., Swaab D., Verhaagen J., Bossers K. (2013). Phenotypic Characterization of Retinoic Acid Differentiated SH-SY5Y Cells by Transcriptional Profiling. PLoS ONE.

[32] Datki Z., Juhász A., Gálfi M., Soós K., Papp R., Zádori D., Penke B. (2003). Method for measuring neurotoxicity of aggregating polypeptides with the MTT assay on differentiated neuroblastoma cells. Brain Res. Bull..

[33] Delenclos M., Burgess J.D., Lamprokostopoulou A., Outeiro T.F., Vekrellis K., McLean P.J. (2019). Cellular models of alpha-synuclein toxicity and aggregation. J. Neurochem..

[34] Lastres-Becker I., Ulusoy A., Innamorato N.G., Sahin G., Rábano A., Kirik D., Cuadrado A.I. (2012). α-Synuclein expression and Nrf2 deficiency cooperate to aggravate protein aggregation, neuronal death and inflammation in early-stage Parkinson’s disease. Hum. Mol. Genet..

[35] Pandey N., Schmidt R.E., Galvin J.E. (2006). The alpha-synuclein mutation E46K promotes aggregation in cultured cells. Exp. Neurol..

[36] Tiraboschi P., Hansen L.A., Alford M., Sabbagh M.N., Schoos B., Masliah E., Thal L.J., Corey-Bloom J. (2000). Cholinergic dysfunction in diseases with Lewy bodies. Neurology.

[37] Fujishiro H., Umegaki H., Isojima D., Akatsu H., Iguchi A., Kosaka K. (2006). Depletion of cholinergic neurons in the nucleus of the medial septum and the vertical limb of the diagonal band in dementia with Lewy bodies. Acta Neuropathol..

[38] Tozzi A., de Iure A., Bagetta V., Tantucci M., Durante V., Quiroga-Varela A., Costa C., Di Filippo M., Ghiglieri V., Latagliata E.C. (2016). Alpha-Synuclein Produces Early Behavioral Alterations via Striatal Cholinergic Synaptic Dysfunction by Interacting With GluN2D N -Methyl-D-Aspartate Receptor Subunit. Biol. Psychiatry.

[39] Vekrellis K., Stefanis L. (2012). Targeting intracellular and extracellular alpha-synuclein as a therapeutic strategy in Parkinson’s disease and other synucleinopathies. Expert Opin. Ther. Targets.

[40] Paleologou K., Irvine G., El-Agnaf O. (2005). α-Synuclein aggregation in neurodegenerative diseases and its inhibition as a potential therapeutic strategy. Biochem. Soc. Trans..

[41] Brundin P., Dave K.D., Kordower J.H. (2017). Therapeutic approaches to target alpha-synuclein pathology. Exp. Neurol..

[42] Stefanis L., Emmanouilidou E., Pantazopoulou M., Kirik D., Vekrellis K., Tofaris G.K. (2019). How is alpha-synuclein cleared from the cell?. J. Neurochem..

[43] Kakish J., Lee D., Lee J.S. (2015). Drugs That Bind to α-Synuclein: Neuroprotective or Neurotoxic?. ACS Chem. Neurosci..

[44] Allison J.R., Rivers R.C., Christodoulou J.C., Vendruscolo M., Dobson C.M. (2014). A Relationship between the Transient Structure in the Monomeric State and the Aggregation Propensities of α-Synuclein and β-Synuclein. Biochemistry.

[45] Periquet M., Fulga T., Myllykangas L., Schlossmacher M., Feany M.B. (2007). Aggregated -Synuclein Mediates Dopaminergic Neurotoxicity In Vivo. J. Neurosci..

[46] Wu K.-P., Baum J. (2010). Detection of Transient Interchain Interactions in the Intrinsically Disordered Protein α-Synuclein by NMR Paramagnetic Relaxation Enhancement. J. Am. Chem. Soc..

[47] El-Agnaf O.M., Jakes R., Curran M.D., Middleton D., Ingenito R., Bianchi E., Pessi A., Neill D., Wallace A. (1998). Aggregates from mutant and wild-type α-synuclein proteins and NAC peptide induce apoptotic cell death in human neuroblastoma cells by formation of β-sheet and amyloid-like filaments. FEBS Lett..

[48] Srivastava T., Raj R., Dubey A., Kumar D., Chaturvedi R.K., Sharma S.K., Priya S. (2020). Fast kinetics of environmentally induced α-synuclein aggregation mediated by structural alteration in NAC region and result in structure dependent cytotoxicity. Sci. Rep..

[49] Ehrnhoefer D.E.E., Bieschke J., Boeddrich A., Herbst M., Masino L., Lurz R., Engemann S., Pastore A., Wanker E.E. (2008). EGCG redirects amyloidogenic polypeptides into unstructured, off-pathway oligomers. Nat. Struct. Mol. Biol..

[50] Ruzza P., Siligardi G., Hussain R., Marchiani A., Islami M., Bubacco L., Delogu G., Fabbri D., Dettori M.A., Sechi M. (2014). Ceftriaxone Blocks the Polymerization of α-Synuclein and Exerts Neuroprotective Effects in Vitro. ACS Chem. Neurosci..

[51] Jia C., Ma X., Liu Z., Gu J., Zhang X., Li D., Zhang S. (2019). Different Heat Shock Proteins Bind α-Synuclein With Distinct Mechanisms and Synergistically Prevent Its Amyloid Aggregation. Front. Neurosci..

[52] Cox D., Whiten D.R., Brown J.W.P., Horrocks M.H., Gil R.S., Dobson C.M., Klenerman D., van Oijen A.M., Ecroyd H. (2018). The small heat shock protein Hsp27 binds α-synuclein fibrils, preventing elongation and cytotoxicity. J. Biol. Chem..

[53] Tavassoly O., Kakish J., Nokhrin S., Dmitriev O., Lee J.S. (2014). The use of nanopore analysis for discovering drugs which bind to α-synuclein for treatment of Parkinson’s disease. Eur. J. Med. Chem..

[54] Ahmad B., Lapidus L.J. (2012). Curcumin Prevents Aggregation in α-Synuclein by Increasing Reconfiguration Rate. J. Biol. Chem..

[55] Zhu M., Rajamani S., Kaylor J., Han S., Zhou F., Fink A.L. (2004). The Flavonoid Baicalein Inhibits Fibrillation of α-Synuclein and Disaggregates Existing Fibrils. J. Biol. Chem..

[56] Lorenzen N., Nielsen S.B., Yoshimura Y., Vad B.S., Andersen C.B., Betzer C., Kaspersen J.D., Christiansen G., Pedersen J.S., Jensen P.H. (2014). How Epigallocatechin Gallate Can Inhibit α-Synuclein Oligomer Toxicity in Vitro. J. Biol. Chem..

[57] Jiang M., Porat-Shliom Y., Pei Z., Cheng Y., Xiang L., Sommers K., Li Q., Gillardon F., Hengerer B., Berlinicke C. (2010). Baicalein reduces E46K α-synuclein aggregation in vitro and protects cells against E46K α-synuclein toxicity in cell models of familiar Parkinsonism. J. Neurochem..

[58] Mbefo M.K., Fares M.-B., Paleologou K., Oueslati A., Yin G., Tenreiro S., Pinto M., Outeiro T., Zweckstetter M., Masliah E. (2015). Parkinson Disease Mutant E46K Enhances α-Synuclein Phosphorylation in Mammalian Cell Lines, in Yeast, and in Vivo. J. Biol. Chem..

[59] Tiong K.H., Yunus N.A.M., Yiap B.C., Tan E.L., Ismail R., Ong C.E. (2014). Inhibitory Potency of 8-Methoxypsoralen on Cytochrome P450 2A6 (CYP2A6) Allelic Variants CYP2A6*15, CYP2A6*16, CYP2A6*21 and CYP2A6*22: Differential Susceptibility Due to Different Sequence Locations of the Mutations. PLoS ONE.

[60] Krieger E., Koraimann G., Vriend G. (2002). Increasing the precision of comparative models with YASARA NOVA-a self-parameterizing force field. Proteins.

